# Bovine Mammary Gene Expression Profiling during the Onset of Lactation

**DOI:** 10.1371/journal.pone.0070393

**Published:** 2013-08-21

**Authors:** Yuanyuan Gao, Xueyan Lin, Kerong Shi, Zhengui Yan, Zhonghua Wang

**Affiliations:** Shandong Provincial Key Laboratory of Ruminant Nutritional Physiology, College of Animal Science and Veterinary Medicine, Shandong Agricultural University, Taian, China; Wageningen UR Livestock Research, The Netherlands

## Abstract

**Background:**

Lactogenesis includes two stages. Stage I begins a few weeks before parturition. Stage II is initiated around the time of parturition and extends for several days afterwards.

**Methodology/Principal Findings:**

To better understand the molecular events underlying these changes, genome-wide gene expression profiling was conducted using digital gene expression (DGE) on bovine mammary tissue at three time points (on approximately day 35 before parturition (−35 d), day 7 before parturition (−7 d) and day 3 after parturition (+3 d)). Approximately 6.2 million (M), 5.8 million (M) and 6.1 million (M) 21-nt cDNA tags were sequenced in the three cDNA libraries (−35 d, −7 d and +3 d), respectively. After aligning to the reference sequences, the three cDNA libraries included 8,662, 8,363 and 8,359 genes, respectively. With a fold change cutoff criteria of ≥2 or ≤−2 and a false discovery rate (FDR) of ≤0.001, a total of 812 genes were significantly differentially expressed at −7 d compared with −35 d (stage I). Gene ontology analysis showed that those significantly differentially expressed genes were mainly associated with cell cycle, lipid metabolism, immune response and biological adhesion. A total of 1,189 genes were significantly differentially expressed at +3 d compared with −7 d (stage II), and these genes were mainly associated with the immune response and cell cycle. Moreover, there were 1,672 genes significantly differentially expressed at +3 d compared with −35 d. Gene ontology analysis showed that the main differentially expressed genes were those associated with metabolic processes.

**Conclusions:**

The results suggest that the mammary gland begins to lactate not only by a gain of function but also by a broad suppression of function to effectively push most of the cell's resources towards lactation.

## Introduction

Lactating cows are generally dried off simply by stopping the milking process approximately two months before the next parturition. The mammary gland then undergoes an involution process, which is marked by the cessation of secretory activity and the reabsorption of milk residue, followed by a relatively static period. The dry period has been proven important for dairy cows. Omitting or shortening the dry period imposes negative effects on mammary health and milk yield in the next lactation [1]–[3]. The mammary gland does not resume its activity until approximately 2 to 3 weeks before the next parturition, when dramatic changes occur to prepare for profuse milk secretion after parturition. Lactogenesis is defined as the process from the resumption of mammary activity until profuse milk secretion and is divided into two stages [4]. Stage I is the period from the resumption of mammary activity to the time of parturition and is characterized by mammary differentiation, proliferation, and progressive expression of milk protein, as well as the secretion of pre-colostrum. Stage II is initiated around the time of parturition and extends for several days afterwards. This stage is characterized by the closure of the tight junctions between alveolar cells and the formation and secretion of colostrum and milk. The mammary gland is the only organ that experiences regular proliferation and involution cycles after maturity, which makes it an ideal model for the study of organ development. Knowledge of the molecular events driving lactogenesis in dairy cows has contributed not only to the understanding of organ development but also to the development of new technologies in the management and breeding of dairy cattle. To date, knowledge about this aspect of the mammary gland has mainly come from studies using mammary cell lines and genetically modified mice [5]–[7]. Some proteins involved in lactogenesis and their related signaling or metabolic pathways have been identified [8], [9]. It has been suggested that there is no sudden transcriptional switch around the time of parturition. Preparation of the gland for lactation includes modifications to the transcriptional program, but the onset of lactation appears to be primarily controlled by post-transcriptional mechanisms [10]. Lemay et al [11] analyzed the microarray data sets of mammary gland RNA samples collected from FVB mice at 10 time points during mammary development, and the results indicated SAM68 (an RNA-binding transduction protein and a putative regulator of mRNA splicing, transduction and nuclear export) to be an important post-transcriptional regulator of both milk secretion and mammary cell survival during lactation. Finucane et al [12] studied the molecular events in stage II of lactogenesis using Affymetrix GeneChip Bovine Genome Array, and found that genes associated with cell cycle and proliferation were downregulated. It was consistent with the result of Sorensen et al [13], which indicated that the mammary glands of cows proliferated mainly in late pregnancy and almost ceased proliferating after parturition. However, in dairy goats, mammary growth continued into early lactation, peaking at day 5 of lactation [14]. In addition, Suchyta et al [15] compared the microarray generated transcript profiles of liver, spleen, thymus, adrenal, ileum, and lymph tissues collected from a 3 month old Holstein steer and of mammary tissues collected from pre-pubertal and post-pubertal Holstein heifers, and identified a putative set of 16 genes being preferentially expressed in the developing mammary gland. In stage II of lactogenesis, the mammary gland undergoes a set of developmental processes that lead to the secretion of colostrum, and then milk. The major compositions of milk are lactose, protein and fat. Bionaz and Loor [9], [16] evaluated the expression of 44 genes involved in bovine mammary milk protein synthesis and 45 genes involved in milk fat synthesis via quantitative PCR. The results of those studies supported a pivotal role for the concerted action of PPARG, PPARGC1A, and INSIG1 in the regulation of milk fat synthesis and a central role of amino acid and glucose transporters and insulin signaling through mTOR in the regulation of protein synthesis in the bovine mammary gland.

Currently, little is known concerning the molecular events underlying lactogenesis. Comparisons of the transcriptomes of pre- and post-parturition mammary glands of dairy cows by Kiera et al [12], provided only some insights into the molecular events of stage II. To gain information on what occurs during stage I, the transcriptomes of the relatively static mammary gland and that of stage I should be compared. Therefore, mammary gland samples from Holstein cows at approximately −35 d, −7 d and +3 d relative to parturition were collected for the present study. Deep sequencing of the transcriptomes in these samples was performed using the newly developed digital gene expression (DGE) method, which was thought to be more reliable, repeatable, and precise compared to previous microarray technology [17].

## Materials and Methods

### Ethics Statement

Our study had been approved by the Institutional Animal Care and Use Committee of Shandong Agricultural University and performed in accordance with the “Guidelines for Experimental Animals” of the Ministry of Science and Technology (Beijing, China).

### Mammary tissue collection

Mammary gland samples were collected from 18 Holstein cows reared in dairy farm No. 1 of the Jiabao dairy company, Jinan, China. They were all in the second parity with the same age. There were 6 cows in each group. Mammary gland tissue samples were collected from cows at three different time-points wherein distinct differences exist in the internal status of the gland: one, 35±2 days before parturition (−35 d) which is a totally dry period, two, 7±2 days before parturition (−7 d) characterized by accelerated cell proliferation and pre-colostrum secretion (stage I of lactogenesis) and three, 3 days after parturition (+3 d) characterized by colostrum production (stage II of lactogenesis). Mammary biopsy was performed using Bard Magnum biopsy system (Bard Peripheral Vascular, Inc., Tempe, AZ, US). The collected samples were immediately frozen and stored in liquid nitrogen until further analysis.

### RNA isolation

To reduce the number of mammary samples needed for DGE analysis, samples from cows in the same group were pooled together for RNA isolation. Total RNA was extracted with Trizol reagent (Invitrogen, Carlsbad, CA, USA) in accordance with the manufacturer's instructions. The quantity and quality of the extracted RNA was determined using a spectrophotometer (Biophotometer Plus, Eppendorf, Germany), with RNA quality being evaluated by the absorbance ratio at 260 nm/280 nm.

### High-throughput sequencing

High-throughput sequencing of mRNA was performed on an Illumina HiSeq 2000 platform. All sample preparation and sequencing procedures were executed according to the manufacturer's instructions (Illumina, Inc., San Diego, CA, USA). Briefly, mRNA from 6 µg of total RNA extracted from samples at each time point was purified by adsorbing to Oligo (dT) magnetic beads, and then use Oligo (dT) as primer to synthesize the first and second-strand cDNA. The bead-bound cDNA was digested with restriction enzyme NlaIII, which recognized and cut off the CATG sites. The fragments apart from the 3′ cDNA fragments connected to Oligo (dT) beads were washed away and the Illumina adaptor 1 was ligated to the sticky 5′ end of the digested bead-bound cDNA fragments. The junction of Illumina adaptor 1 and CATG site was the recognition site of Mmel, which was a type of Endonuclease with separated recognition sites and digestion sites. It cut at 17 bp downstream of the CATG site, producing tags with adaptor 1. After removing 3′ fragments with magnetic beads precipitation, Illumina adaptor 2 was ligated to the 3′ ends of tags, acquiring tags with different adaptors of both ends to form a tag library. After 15 cycles of linear PCR amplification, 105 bp fragments are purified by 6% TBE PAGE Gel electrophoresis. After denaturation, the single-stranded cDNA was anchored on Illumina flowcells of the Illumina HiSeq 2000 system and sequenced. Raw data (tag sequences) were deposited in NCBI's Gene Expression Omnibus (GEO) database under submission number GSE44796.

### Tag mapping

For the raw data, we filtered adaptor sequences, low quality tags (tags with unknown nucleotides N), empty reads and tags that were too short or too long, and tags with only one copy to get clean tags.

A virtual library containing all the possible CATG+17 bases length sequences of the bovine gene sequences were constructed by BGI-shenzhen [17]–[19], using Bos taurus UMD 3.1 [20]. All clean tags were mapped to the reference sequences, and only upto 1-bp mismatch was considered. Clean tags that mapped to reference sequences from multiple genes were filtered out. The remaining clean tags were designated as unambiguous clean tags. The number of unambiguous clean tags for each gene was calculated and then normalized to TPM (number of transcripts per million clean tags).

### Screening of differentially expressed genes

A rigorous algorithm has been developed to identify genes expressed differentially between samples. Because every gene's expression occupies only a small part of the library, p(x) was within the Poisson distribution. The probability of a given gene being expressed equally between two samples can be calculated with the following equation [21]:
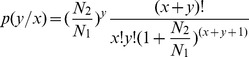
where the total number of clean tags in sample 1 is N_1_, the total number of clean tags in sample 2 is N_2_, and there are x tags in sample 1 and y tags in sample 2 for gene A. False Discovery Rate (FDR) analysis was applied to determine the threshold P value in multiple tests and analyses [22]. The significance of the difference in gene expression was judged using a threshold of FDR≤0.001 and fold-change value ≥2 (|log2 Ratio|≥1).

### Sequencing data analysis

The selected genes with significant modification in their expression were subjected to further analysis, which were GO enrichment analysis, pathway enrichment analysis and functional annotation clustering.

GO enrichment analysis of functional significance applies hypergeometric test to map all differentially expressed genes (DEGs) to terms in Gene Ontology (GO) database (Version 1.1.1631) [23], looking for significantly enriched GO terms in DEGs comparing to the genome background. The calculating formula is [24]:
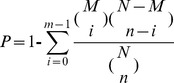
where N is the number of all genes with GO annotation; n is the number of DEGs in N; M is the number of all genes that are annotated to the certain GO terms; m is the number of DEGs in M. After Bonferroni correction, GO terms with corrected-p value ≤0.05 were significantly enriched in differentially expressed genes.

Pathway enrichment analysis based on Kyoto Encyclopedia of Genes and Genomes (KEGG) database (Version 58) can identify significantly enriched biological pathways in DEGs compared with the whole genome background [25]. Enriched pathways were calculated using the same formula as that in GO analysis. Here N is the number of all genes with KEGG annotation, n is the number of DEGs in N, M is the number of all genes annotated in specific pathways, and m is the number of DEGs in M. Pathways with a Q value ≤0.05 were significantly enriched in differentially expressed genes.

The differentially expressed genes detected in stage I and stage II of lactogenesis were further analyzed using DAVID functional annotation clustering tool [26]. This tool can provide a look at the internal relationships of the clustered terms.

### Quantitative real-time PCR (qRT-PCR)

QRT-PCR analysis was used to verify the DGE results. The RNA samples used for the qRT-PCR assay were both the same as for the DGE experiments and independent RNA extractions from biological replicates. 13 candidate genes were selected and detected using qRT-PCR, including caseinβ, CIDEA, GLYCAM, BGN, ELL3, PAH, GP2, LOC525947, SLC14A1, SST, PNMT, caseinα-S2 and integrinβ. qRT-PCR was performed according to the TaKaRa manufacturer specifications (TaKaRa SYBR® PrimeScript™ RT-PCR Kit, Dalian, China). SYBR Green PCR cycling was denatured using a program of 95°C for 10 s, and 40 cycles of 95°C for 5 s and 60°C for 40 s, and performed on an ABI 7500 instrument (Applied Biosystems, Foster City, CA, USA). Data were reported as values normalized to the housekeeping gene β-actin, and they were subjected to one-way ANOVA analysis using Statistical Analysis Systems statistical software package (Version 8e, SAS Institute, Cary, NC, USA). Means were considered significantly different at p<0.05.

## Results

### Analysis of the three cDNA libraries

**Figure 1:**
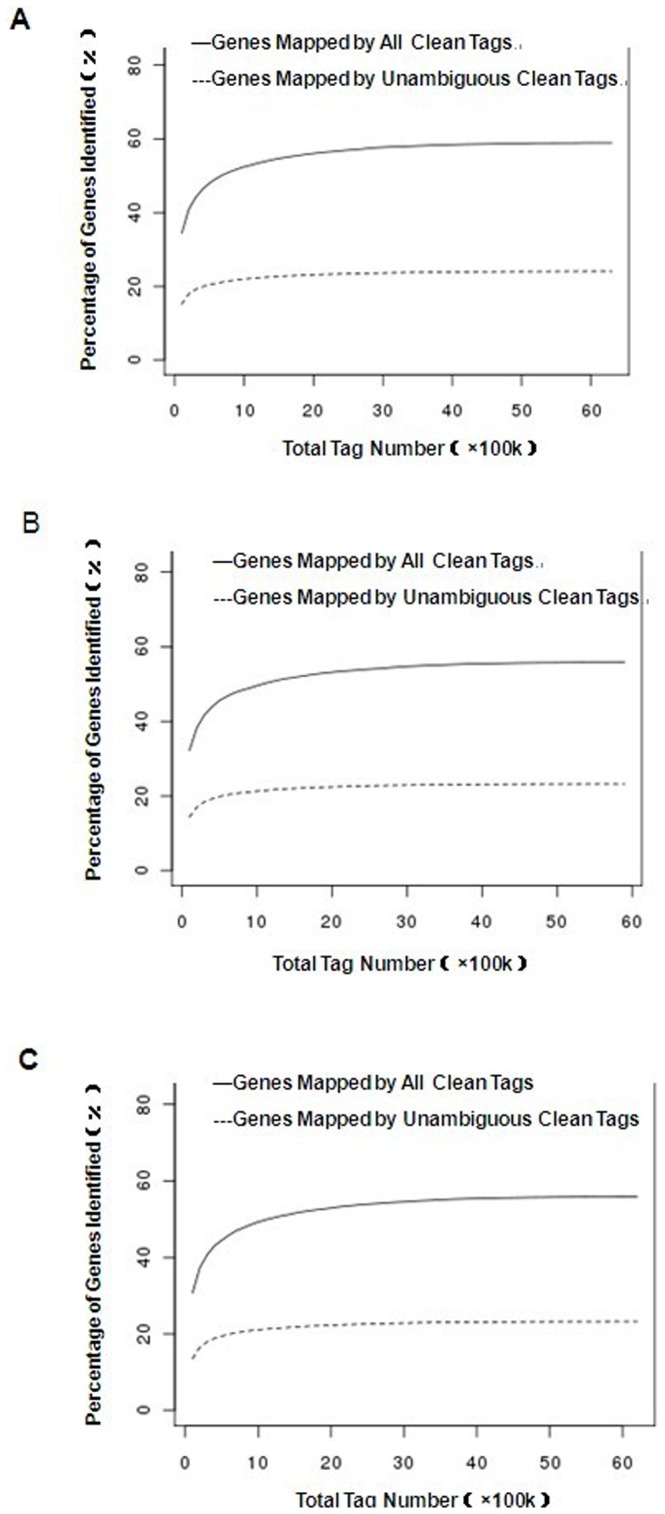
Saturation analysis of −35 d (A), −7 d (B) and +3 d (C) libraries sequencing. “—” represents genes mapped by all clean tags. “---” represents genes mapped by unambiguous clean tags. The number of detected genes continued increasing as the total tag number increased. When the number of total tags reached 2M or more, the number of detected genes almost ceased increasing.

Characterization of the three cDNA libraries (−35 d, −7 d and +3 d) was listed in Table 1. Sequencing depths of 6,203,209, 5,857,755 and 6,115,741 tags were identified in the three libraries, which included 192,928, 170,427 and 168,233 distinct tags, respectively. As the copy number of the tags increased, the number of distinct tags decreased. Saturation analysis was performed to check whether the number of detected genes continues to increase as the number of tags sequenced (total tag number) increases. The results were shown in Figure 1. When the number of sequenced tags reached 2M or more, the number of detected genes almost ceased increasing. There were 6.2M, 5.8M and 6.1M tags in the three libraries; thus, all three were sequenced to saturation, producing a full representation of the transcripts present in each of the three stages.

**Table 1:** Distribution of the tags sequenced from the three libraries.

	Tag library		
	−35 d	−7 d	+3 d
Total tag number	6,203,209	5,857,755	6,115,741
Total clean tag number	192,928	170,427	168,233
Distinct clean tag number	476,719	429,231	430,516
Tag copy number <2	273,198	250,858	254,875
Tag copy number ≥2 (clean tags)	192,928 (100%)	170,427 (100%)	168,233 (100%)
Tag copy number >5	79,694 (41.31%)	67,230 (39.45%)	63,337 (37.65%)
Tag copy number >10	50,966 (26.42%)	42,877 (25.16%)	39,889 (23.71%)
Tag copy number >20	31,557 (16.36%)	26,947 (15.81%)	24,676 (14.67%)
Tag copy number >50	15,682 (8.13%)	14,034 (8.23%)	12,451 (7.40%)
Tag copy number >100	8,427 (4.37%)	7,864 (4.61%)	6,693 (3.98%)

### Analysis of tag annotation

**Figure 2:**
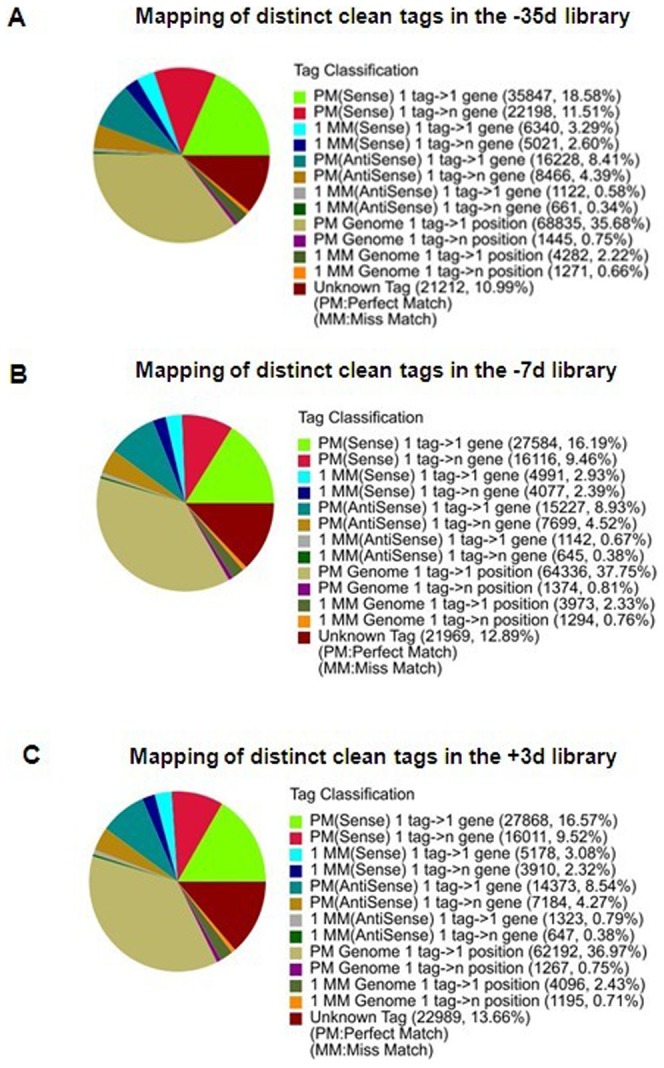
Summary of the distinct tag-to-gene mapping data in the three libraries.

A reference database of the bovine genome includes 35,945 transcripts [20]. A total of 193,547 reference tags with 94,396 (48.77%) unambiguous reference tags were obtained. All clean tags in the three cDNA libraries were mapped to the reference tags, and the results were shown in Figure 2. There were 59,537 (30.86%), 48,944 (28.72%) and 48,742 (28.98%) distinct tags matched to the reference genes in the three libraries (−35 d, −7 d and +3 d), respectively. Among these tags, there were 8.99%, 9.60% and 9.33% tags, respectively, matched to the anti-sense strand of the genes, suggesting that the antisense strand of these genes also had transcripts and that these genes might therefore have sense-antisense regulation. The unmatched tags were then mapped to the bovine genome, and 37.90%, 40.08% and 39.40% of tags, respectively, were matched to the genomic sequences in the three libraries. These tags might represent non-annotated genes or could possibly be derived from intergenic regions not encoding any transcripts.

### Detection of differentially expressed genes and GO enrichment analysis

**Figure 3:**
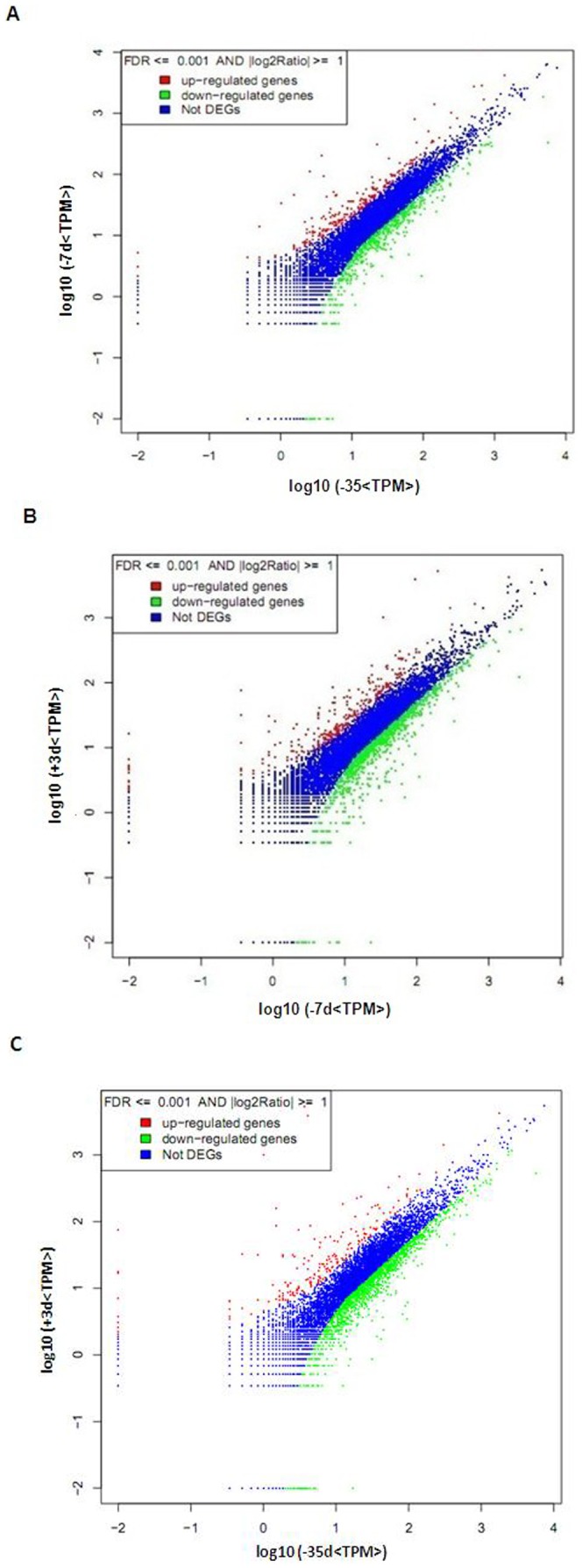
Pairwise comparisons of the level of gene expression between libraries. To compare the level of gene expression between two libraries, each library was normalized to TPM. (A) Differentially expressed genes at −7 d compared with −35 d. (B) Differentially expressed genes at +3 d compared with −7 d. (C) Differentially expressed genes at +3 d compared with −35 d. Red dots represent upregulated transcripts. Green dots represent downregulated transcripts. Blue dots represent transcripts that did not change significantly. The parameters FDR≤0.001 and |log2 Ratio|≥1 were used as the threshold to judge the significance of the difference in gene expression. See supplementary data 1, 2 and 3 for details of these differentially expressed genes.

**Figure 4:**
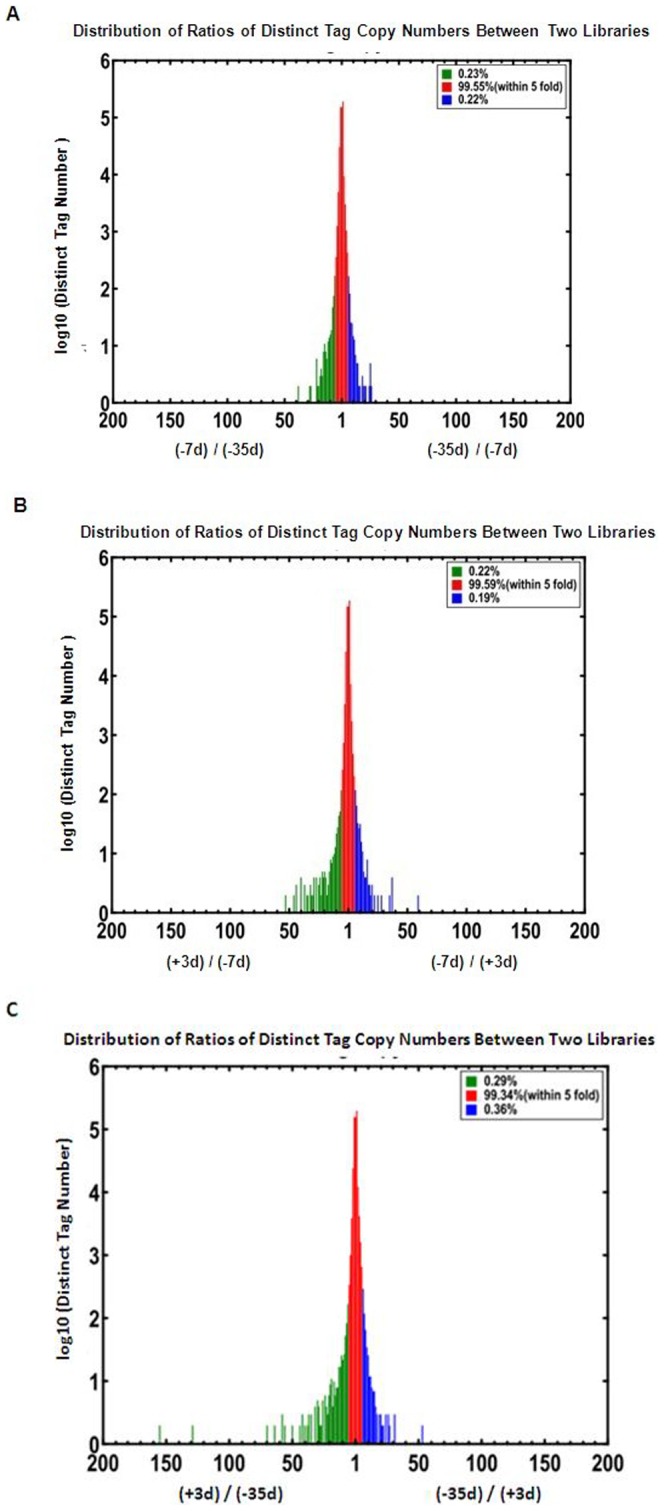
Differentially expressed tags at the onset of lactation. (A) Differentially expressed tags at −7 d compared with −35 d. (B) Differentially expressed tags at +3 d compared with −7 d. (C) Differentially expressed tags at +3 d compared with −35 d. The “x” represents the fold-change of differentially expressed unique tags. The “y” axis represents the number of unique tags (log10). The red region represents differentially accumulating unique tags with a 5-fold difference between libraries. The green and blue regions represent unique tags that are upregulated and downregulated for more than 5 fold.

At the cutoff criteria of FDR≤0.001 and |log2 Ratio|≥1, many genes were differentially expressed; these results were shown in Figure 3. The details of these differentially expressed genes were supplied in Excel S1, S2 and S3. A total of 812 genes were significantly differentially expressed at −7 d compared with −35 d (stage I), accounting for 9.70% of transcripts in the −7 d cDNA library. There were 234 (28.80%) genes upregulated (in red) and 578 (71.20%) genes downregulated (in green). The number of genes having greater than a five-fold difference accounted for 0.45% of the total differentially expressed genes (Figure 4). A total of 1,189 (14.20%) genes were significantly differentially expressed, with 274 (23.00%) genes upregulated and 915 (77.00%) genes downregulated at +3 d compared with −7 d (stage II). The number of genes having greater than a five-fold difference accounted for 0.41% of the total differentially expressed genes. Moreover, there were 1,672 genes significantly differentially expressed at +3 d compared with −35 d. There were 209 (12.5%) upregulated genes, and 1,463 (87.5%) downregulated genes. Among those genes, there were 11 (0.65%) genes having greater than a five-fold difference.

The differentially expressed genes were analyzed in the context of gene ontology (GO) biological processes. This analysis revealed that the upregulated genes in DEGs were mainly associated with the cell cycle and lipid metabolism, and the downregulated genes were mainly associated with the immune response and biological adhesion at −7 d compared with −35 d. And the upregulated genes in DEGs at +3 d compared with −7 d were mainly associated with the immune response, and the downregulated genes were mainly associated with the cell cycle phase. The most significantly enriched biological processes in DEGs were metabolic processes at +3 d compared with −35 d.

Moreover, during the onset of lactation, some genes were completely turned on or off. There were 305 genes turned on at −7 d compared with −35 d. Gene ontology analysis showed that these genes were associated with signal transduction (GABRB1; GPRC5A; GPR107; RAB9B), transport (SLC5A1; SLC13A3; KCNC1; KCNAB3; SLC38A5), the cell cycle (CDCA5; HORMAD1; FSD1; MPHOSPH9), and other processes. In addition, 12 genes encoding hypothetical proteins (LOC100335323, LOC100336058, LOC100335520) were also identified in stage I of lactogenesis. 604 genes were turned off at −7 d compared with −35 d. Gene ontology analysis showed that these genes were associated with ion transport (SLC26A2; SCN4B; TMEM163; SLC5A12; SLC13A4; SLC39A11; SLCO2A1) and protein and lipid metabolism (PREP; caspase-15; LPGAT1; PLCB4; EPT1). There were 421 genes turned on at +3 d compared with −7 d, including genes involved in transport (SLC5A2; SLC26A2; KCNH2), the immune response (MICB; CCL28; IL8), and signal transduction (PIK3R3; AKAP4). 425 genes were turned off at +3 d compared with −7 d, including cell cycle-related genes (CDC25C; SKA1; FSD1) and signal transduction genes (GABRB1; GPRC5A; GPR37).

### Pathway enrichment analysis of differentially expressed genes

After pathway enrichment analysis, the top 10 significantly enriched pathways were listed in Tables 2, 3 and 4. The results suggested that many of the pathways enriched in DEGs were associated with the immune response at −7 d compared with −35 d, while many of the pathways enriched in DEGs were associated with the cell cycle and metabolism at +3 d compared with −7 d. And the most enriched pathway in DEGs was metabolic pathways at +3 d compared with −35 d.

**Table 2:** Top 10 significantly enriched pathways at −7 d compared with −35 d.

Pathway	DEGs with pathway annotation	Q value	Pathway ID
Phagosome	41 (6.23%)	2.87e-10	ko04145
Complement and coagulation cascades	22 (3.34%)	3.12e-04	ko04610
Lysosome	23 (3.5%)	3.12e-04	ko04142
Rheumatoid arthritis	18 (2.74%)	5.85e-04	ko05323
Hematopoietic cell lineage	17 (2.58%)	1.60e-03	ko04640
*Staphylococcus aureus* infection	19 (2.89%)	1.71e-03	ko05150
Cell adhesion molecules (CAMs)	26 (3.95%)	1.84e-03	ko04514
Metabolic pathways	90 (13.68%)	4.16e-03	ko01100
PPAR signaling pathway	15 (2.28%)	9.70e-03	ko03320
Bladder cancer	9 (1.37%)	9.70e-03	ko05219

**Table 3:** Top 10 significantly enriched pathways at +3 d compared with −7 d.

Pathway	DEGs with pathway annotation	Q value	Pathway ID
Metabolic pathways	172 (18.03%)	3.74e-15	ko01100
Proteasome	18 (1.89%)	6.43e-10	ko03050
DNA replication	15 (1.57%)	5.50e-08	ko03030
Base excision repair	14 (1.47%)	3.04e-06	ko03410
Cell cycle	30 (3.14%)	6.36e-06	ko04110
Pyrimidine metabolism	23 (2.41%)	4.32e-05	ko00240
Purine metabolism	33 (3.46%)	5.13e-05	ko00230
Oxidative phosphorylation	25 (2.62%)	5.90e-05	ko00190
Propanoate metabolism	12 (1.26%)	9.46e-05	ko00640
Mismatch repair	9 (0.94%)	2.17e-04	ko03430

**Table 4:** Top 10 significantly enriched pathways in differentially expressed genes at +3 d compared with −35 d.

Pathway	DEGs with pathway annotation	Q value	Pathway ID
Metabolic pathways	205 (15.67%)	1.32e-11	ko01100
Oxidative phosphorylation	38 (2.91%)	2.35e-08	ko00190
DNA replication	16 (1.22%)	4.23e-07	ko03030
Proteasome	17 (1.3%)	4.23e-07	ko03050
Base excision repair	16 (1.22%)	2.99e-06	ko03410
Cell cycle	35 (2.68%)	2.16e-05	ko04110
Phagosome	48 (3.67%)	2.16e-05	ko04145
Parkinson's disease	35 (2.68%)	2.95e-05	ko05012
Nucleotide excision repair	16 (1.22%)	3.78e-04	ko03420
Huntington's disease	45 (3.44%)	5.80e-04	ko05016

### Analysis of milk protein genes and lipogenic genes

The expression level of many milk protein genes increased significantly during stage I of lactogenesis and kept increasing during stage II. These genes include casein kappa (CSN3), lactalbumin alpha (LALBA), casein beta (CSN2), casein alpha s1 (CSN1S1), and casein alpha-S2 (CSN1S2). The fold changes of these genes were listed in Table 5. Furthermore, many lipogenic genes changed significantly at the onset of lactation. These genes were associated with mammary fatty acids uptake from the blood (LPL, CD36), intracellular fatty acids trafficking (FABP3), long-chain (ACSL1) and short-chain (ACSS2) intracellular fatty acids activation, de novo fatty acids synthesis (ACACA, FASN), desaturation (SCD), triacylglycerol synthesis (AGPAT6, GPAM), lipid droplet formation (BTN1A1, XDH), ketone body utilization (BDH1), and transcription regulation (INSIG1, PPARGC1A). The fold changes of these genes were listed in Table 6.

**Table 5:** Expression changes of milk protein genes during the two stages of lactogenesis.

Gene	Description	TPM (−35 d)	TPM (−7 d)	TPM (3 d)	log_2_ Ratio (−7/−35)	log_2_ Ratio (+3/−7)
gi|31341749|ref|NM_174528.2|	Casein alpha –S2 (CSN1S2)	22.49	304.12	10389.3	3.76	5.09
gi|31342165|ref|NM_174378.2|	Lactalbumin alpha (LALBA)	3.72	198.63	5224.76	5.74	4.72
gi|31341348|ref|NM_181029.2|	Casein alpha s1 (CSN1S1)	578.92	10599.41	80620.84	4.19	2.93
gi|31341343|ref|NM_181008.2|	Casein beta (CSN2)	735.32	12496.16	64462.97	4.09	2.37
gi|27881411|ref|NM_174294.1|	Casein kappa (CSN3)	1390.84	4109.25	13441.57	1.56	1.71

Note: TPM (Transcripts Per Million clean tags) is a standardized indicator that specifies the number of transcript copies in every one million clean tags.

**Table 6:** Expression changes of lipogenic genes during the two stages of lactogenesis.

Gene	Description	TPM (−35 d)	TPM (−7 d)	TPM (+3 d)	log_2_ Ratio (−7/−35)	log_2_ Ratio (+3/−7)
gi|115497163|ref|NM_001075120.1|	lipoprotein lipase (LPL)	35.17	38.26	172.26	0.12	2.17*
gi|31343049|ref|NM_174010.2|	CD36 molecule (thrombospondin receptor) (CD36)	156.9	77.24	272.33	−1.02*	1.82*
gi|31342355|ref|NM_174313.2|	fatty acid binding protein 3, muscle and heart (mammary-derived growth inhibitor) (FABP3)	1.52	46.31	156.52	4.93*	1.76*
gi|155372064|ref|NM_001101169.1|	solute carrier family 27 (fatty acid transporter) member 6 (SLC27A6)	9.64	36.12	50.98	1.91*	0.50
gi|115497269|ref|NM_001076085.1|	acyl-CoA synthetase long-chain family member 1 (ACSL1)	96.37	174.68	115.64	0.86	−0.60
gi|157427805|ref|NM_001105339.1|	acyl-CoA synthetase short-chain family member 2 (ACSS2)	30.27	78.85	32.33	1.38*	−1.29*
gi|31342550|ref|NM_174224.2|	acetyl-CoA carboxylase alpha (ACACA)	1.86	2.5	2.74	0.43	0.13
gi|60592789|ref|NM_001012669.1|	fatty acid synthase (FASN)	660.42	2804.46	652.95	2.09*	−2.10*
gi|148540093|ref|NM_173959.4|	stearoyl-CoA desaturase (delta-9-desaturase) (SCD)	74.73	796.32	471.62	3.41*	−0.76
gi|139948314|ref|NM_001083669.1|	1-acylglycerol-3-phosphate O-acyltransferase 6 (lysophosphatidic acid acyltransferase, zeta) (AGPAT6)	41.59	32.72	44.48	−0.35	0.44
gi|59676567|ref|NM_001012282.1|	glycerol-3-phosphate acyltransferase, mitochondrial (GPAM)	77.27	113.53	107.77	0.56	−0.075
gi|148232524|ref|NM_205793.2|	diacylglycerol O-acyltransferase 2 (DGAT2)	12.17	29.5	2.57	1.28*	−3.52*
gi|31341801|ref|NM_174508.2|	butyrophilin subfamily 1 member A1 (BTN1A1)	4.4	42.55	85.87	3.27*	1.01*
gi|31343144|ref|NM_173972.2|	xanthine dehydrogenase (XDH)	299.44	827.07	1390.57	1.47*	0.75
gi|77736146|ref|NM_001034600.1|	3-hydroxybutyrate dehydrogenase, type 1 (BDH1)	0.51	2.5	2.91	2.29	0.22
gi|118150923|ref|NM_001077909.1|	insulin induced gene 1 (INSIG1)	40.24	94.94	65	1.24*	−0.55
gi|164519007|ref|NM_001113302.1|	sterol regulatory element binding transcription factor 1 (SREBF1)	57.99	147.32	92.03	1.35*	−0.68

Note:

* represents significant difference at the cutoff criteria of FDR≤0.001 and |log2 Ratio|≥1.

### Analysis of differentially expressed genes throughout the onset of lactation

**Figure 5:**
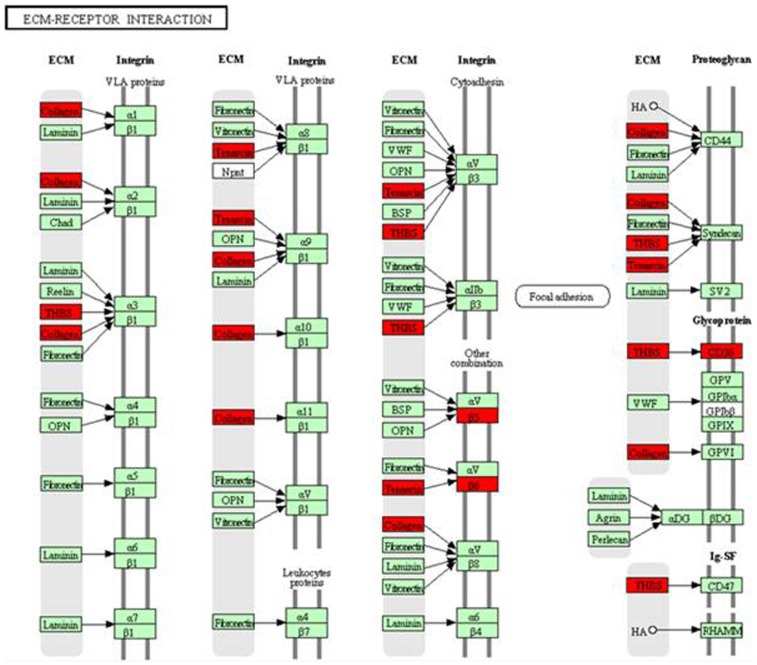
The ECM-Receptor interaction pathway is transcriptionally regulated throughout the onset of lactation. Red nodes represent genes downregulated during stage I of lactogenesis and then upregulated during stage II of lactogenesis.

There were 231 genes differentially expressed in the two stages of lactogenesis. Among these genes, 92 genes were downregulated in stage I and upregulated in stage II, 111 genes were upregulated in stage I and downregulated in stage II, and only 11 genes continued to increase in both stages. After analysis of functional annotation clustering, 92 genes fell into 24 clusters. Each term in Cluster 4 was associated with the immune response, and each in cluster two was associated with the extracellular matrix. The top 5 enriched clusters were listed in Table 7. After pathway enrichment analysis, the ECM-Receptor interaction was enriched (Figure 5). In addition, functional annotation clustering of the 111 genes that were upregulated initially and then downregulated fell into 19 clusters; the top 5 enriched clusters were listed in Table 8. We can find that all terms in Cluster 1 were associated with cell cycle.

**Table 7:** Functional annotation clustering of genes downregulated in stage I and then upregulated in stage II of lactogenesis.

Category	Terms	N	Percent(%)	P-Value
**Cluster 1**	**Enrichment Score: 12.80**			
GOTERM_CC_FAT	GO:0005576∼extracellular region	36	41.38	2.88E-17
UP_SEQ_FEATURE	signal peptide	37	42.53	6.67E-15
SP_PIR_KEYWORDS	signal	37	42.53	1.07E-14
SP_PIR_KEYWORDS	Secreted	29	33.33	9.55E-14
SP_PIR_KEYWORDS	disulfide bond	32	36.78	9.52E-13
UP_SEQ_FEATURE	glycosylation site:N-linked (GlcNAc…)	34	39.08	3.16E-12
SP_PIR_KEYWORDS	glycoprotein	35	40.23	6.54E-12
UP_SEQ_FEATURE	disulfide bond	29	33.33	9.64E-11
**Cluster 2**	**Enrichment Score: 5.50**			
GOTERM_CC_FAT	GO:0044421 extracellular region part	20	22.99	8.79E-10
GOTERM_CC_FAT	GO:0031012 extracellular matrix	11	12.64	1.96E-06
SP_PIR_KEYWORDS	extracellular matrix	7	8.05	1.36E-04
GOTERM_CC_FAT	GO:0005578 proteinaceous extracellular matrix	8	9.20	4.14E-04
**Cluster 3**	**Enrichment Score: 5.46**			
INTERPRO	IPR014716:Fibrinogen, alpha/beta/gamma chain, C-terminal globular, subdomain 1	6	6.90	2.87E-07
INTERPRO	IPR002181:Fibrinogen, alpha/beta/gamma chain, C-terminal globular	6	6.90	7.05E-07
SMART	SM00186:FBG	6	6.90	3.55E-06
UP_SEQ_FEATURE	domain:Fibrinogen C-terminal	4	4.60	2.02E-04
**Cluster 4**	**Enrichment Score: 3.68**			
GOTERM_BP_FAT	GO:0002526 acute inflammatory response	7	8.05	5.29E-07
GOTERM_BP_FAT	GO:0006954 inflammatory response	8	9.20	8.54E-06
GOTERM_BP_FAT	GO:0002541 activation of plasma proteins involved in acute inflammatory response	5	5.75	8.59E-06
GOTERM_BP_FAT	GO:0006956 complement activation	5	5.75	8.59E-06
GOTERM_BP_FAT	GO:0006957 complement activation, alternative pathway	4	4.60	1.21E-05
SP_PIR_KEYWORDS	complement alternate pathway	4	4.60	2.82E-05
GOTERM_BP_FAT	GO:0006959 humoral immune response	5	5.75	3.19E-05
GOTERM_BP_FAT	GO:0009611 response to wounding	9	10.34	3.22E-05
GOTERM_BP_FAT	GO:0045087 innate immune response	6	6.90	7.05E-05
GOTERM_BP_FAT	GO:0051605 protein maturation by peptide bond cleavage	5	5.75	1.48E-04
GOTERM_BP_FAT	GO:0050778 positive regulation of immune response	6	6.90	2.14E-04
GOTERM_BP_FAT	GO:0002253 activation of immune response	5	5.75	3.40E-04
SP_PIR_KEYWORDS	innate immunity	5	5.75	3.93E-04
GOTERM_BP_FAT	GO:0016485 protein processing	5	5.75	5.02E-04
KEGG_PATHWAY	bta04610:Complement and coagulation cascades	5	5.75	5.36E-04
GOTERM_BP_FAT	GO:0051604 protein maturation	5	5.75	6.23E-04
GOTERM_BP_FAT	GO:0006952 defense response	8	9.20	0.00
GOTERM_BP_FAT	GO:0048584 positive regulation of response to stimulus	6	6.90	0.00
GOTERM_BP_FAT	GO:0002252 immune effector process	5	5.75	0.00
GOTERM_BP_FAT	GO:0002684 positive regulation of immune system process	6	6.90	0.00
SP_PIR_KEYWORDS	immune response	5	5.75	0.00
GOTERM_BP_FAT	GO:0006955 immune response	7	8.05	0.02
GOTERM_BP_FAT	GO:0006508 proteolysis	9	10.34	0.07
**Cluster 5**	**Enrichment Score: 2.84**			
KEGG_PATHWAY	bta04512:ECM-receptor interaction	7	8.05	2.30E-06
INTERPRO	IPR013032:EGF-like region, conserved site	9	10.34	7.39E-06
INTERPRO	IPR006210:EGF-like	7	8.05	1.20E-04
SMART	SM00181:EGF	7	8.05	6.99E-04
INTERPRO	IPR013111:EGF, extracellular	4	4.60	0.00
GOTERM_BP_FAT	GO:0007155 cell adhesion	9	10.34	0.00
GOTERM_BP_FAT	GO:0022610 biological adhesion	9	10.34	0.00
KEGG_PATHWAY	bta04510:Focal adhesion	6	6.90	0.00
SP_PIR_KEYWORDS	cell adhesion	6	6.90	0.00
GOTERM_BP_FAT	GO:0007160 cell-matrix adhesion	3	3.45	0.03
GOTERM_BP_FAT	GO:0031589 cell-substrate adhesion	3	3.45	0.03
INTERPRO	IPR002035:von Willebrand factor, type A	3	3.45	0.04
SMART	SM00327:VWA	3	3.45	0.08

**Table 8:** Functional annotation clustering of genes upregulated during stage I and then downregulated during stage II of lactogenesis.

Category	Term	N	Percent(%)	P-Value
**Cluster 1**	**Enrichment Score: 2.48**			
GOTERM_BP_FAT	GO:0007049 cell cycle	11	10.68	7.85E-06
GOTERM_BP_FAT	GO:0022402 cell cycle process	9	8.74	2.51E-05
GOTERM_BP_FAT	GO:0022403 cell cycle phase	8	7.77	3.66E-05
GOTERM_BP_FAT	GO:0000279 M phase	7	6.80	9.29E-05
GOTERM_BP_FAT	GO:0000278 mitotic cell cycle	7	6.80	1.03E-04
GOTERM_BP_FAT	GO:0000280 nuclear division	6	5.83	1.77E-04
GOTERM_BP_FAT	GO:0007067 mitosis	6	5.83	1.77E-04
SP_PIR_KEYWORDS	cell division	7	6.80	2.23E-04
GOTERM_BP_FAT	GO:0000087 M phase of mitotic cell cycle	6	5.83	2.24E-04
GOTERM_BP_FAT	GO:0048285 organelle fission	6	5.83	2.37E-04
GOTERM_BP_FAT	GO:0051301 cell division	6	5.83	0.00
GOTERM_CC_FAT	GO:0000775 chromosome, centromeric region	5	4.85	0.00
SP_PIR_KEYWORDS	cell cycle	7	6.80	0.00
SP_PIR_KEYWORDS	mitosis	5	4.85	0.00
GOTERM_CC_FAT	GO:0005694 chromosome	8	7.77	0.00
GOTERM_CC_FAT	GO:0044427 chromosomal part	7	6.80	0.01
GOTERM_CC_FAT	GO:0005819 spindle	4	3.88	0.01
GOTERM_CC_FAT	GO:0015630 microtubule cytoskeleton	6	5.83	0.03
GOTERM_BP_FAT	GO:0007017 microtubule-based process	4	3.88	0.06
GOTERM_CC_FAT	GO:0000793 condensed chromosome	3	2.91	0.06
SP_PIR_KEYWORDS	cytoskeleton	5	4.85	0.10
GOTERM_CC_FAT	GO:0043228 non-membrane-bounded organelle	13	12.62	0.18
GOTERM_CC_FAT	GO:0043232 intracellular non-membrane-bounded organelle	13	12.62	0.18
GOTERM_CC_FAT	GO:0005874 microtubule	3	2.91	0.19
GOTERM_CC_FAT	GO:0044430 cytoskeletal part	6	5.83	0.21
GOTERM_CC_FAT	GO:0005856 cytoskeleton	7	6.80	0.30
SP_PIR_KEYWORDS	cytoplasm	10	9.71	0.69
**Cluster 2**	**Enrichment Score: 2.34**			
GOTERM_MF_FAT	GO:0048037∼cofactor binding	9	8.74	3.42E-05
GOTERM_MF_FAT	GO:0050662∼coenzyme binding	5	4.85	0.01
GOTERM_BP_FAT	GO:0055114∼oxidation reduction	6	5.83	0.261
**Cluster 3**	**Enrichment Score: 1.71**			
GOTERM_MF_FAT	GO:0030554∼adenyl nucleotide binding	20	19.42	4.08E-05
GOTERM_MF_FAT	GO:0001883∼purine nucleoside binding	20	19.42	4.63E-05
GOTERM_MF_FAT	GO:0001882∼nucleoside binding	20	19.42	5.00E-05
GOTERM_MF_FAT	GO:0032559∼adenyl ribonucleotide binding	17	16.50	7.63E-04
GOTERM_MF_FAT	GO:0017076∼purine nucleotide binding	20	19.42	8.58E-04
GOTERM_MF_FAT	GO:0005524∼ATP binding	16	15.53	0.00
SP_PIR_KEYWORDS	atp-binding	13	12.62	0.00
GOTERM_MF_FAT	GO:0000166∼nucleotide binding	21	20.39	0.00
GOTERM_MF_FAT	GO:0032553∼ribonucleotide binding	17	16.50	0.01
GOTERM_MF_FAT	GO:0032555∼purine ribonucleotide binding	17	16.50	0.01
INTERPRO	IPR017441:Protein kinase, ATP binding site	6	5.83	0.03
INTERPRO	IPR000719:Protein kinase, core	6	5.83	0.06
SP_PIR_KEYWORDS	nucleotide-binding	12	11.65	0.06
GOTERM_MF_FAT	GO:0004674∼protein serine/threonine kinase activity	5	4.85	0.11
GOTERM_MF_FAT	GO:0004672∼protein kinase activity	6	5.83	0.15
SP_PIR_KEYWORDS	serine/threonine-protein kinase	4	3.88	0.15
GOTERM_BP_FAT	GO:0006468∼protein amino acid phosphorylation	6	5.83	0.18
INTERPRO	IPR008271:Serine/threonine protein kinase, active site	4	3.88	0.19
SMART	SM00220:S_TKc	3	2.91	0.22
INTERPRO	IPR017442:Serine/threonine protein kinase-related	4	3.88	0.24
GOTERM_BP_FAT	GO:0016310∼phosphorylation	6	5.83	0.27
INTERPRO	IPR002290:Serine/threonine protein kinase	3	2.91	0.29
SP_PIR_KEYWORDS	kinase	4	3.88	0.42
GOTERM_BP_FAT	GO:0006793∼phosphorus metabolic process	6	5.83	0.44
GOTERM_BP_FAT	GO:0006796∼phosphate metabolic process	6	5.83	0.44
**Cluster 4**	**Enrichment Score: 1.37**			
GOTERM_BP_FAT	GO:0006259∼DNA metabolic process	7	6.80	0.01
GOTERM_BP_FAT	GO:0006281∼DNA repair	4	3.88	0.06
GOTERM_BP_FAT	GO:0033554∼cellular response to stress	5	4.85	0.07
GOTERM_BP_FAT	GO:0006974∼response to DNA damage stimulus	4	3.88	0.10
**Cluster 5**	**Enrichment Score: 1.28**			
GOTERM_BP_FAT	GO:0008203∼cholesterol metabolic process	3	2.91	0.03
GOTERM_BP_FAT	GO:0016125∼sterol metabolic process	3	2.91	0.04
GOTERM_BP_FAT	GO:0008202∼steroid metabolic process	3	2.91	0.11

### Quantitative real-time PCR (qRT-PCR) confirmation

**Figure 6:**
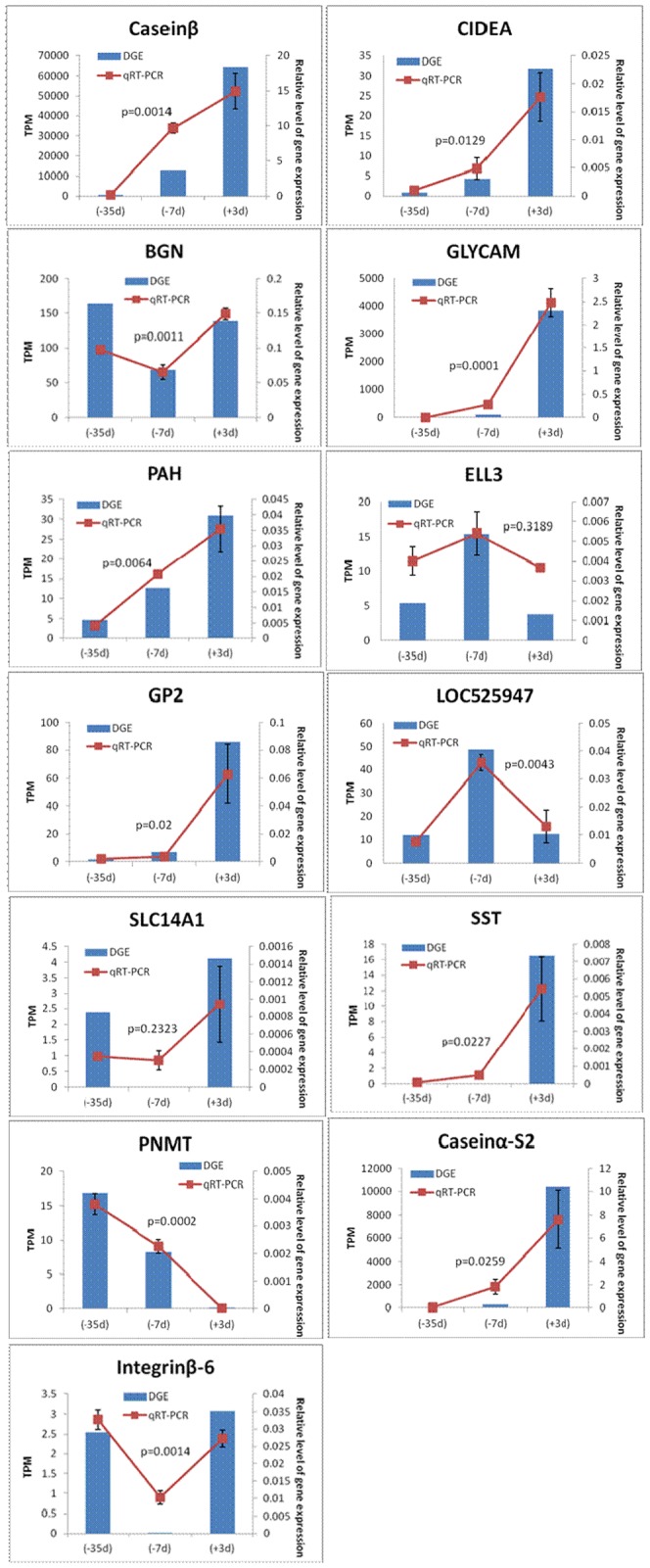
Comparison of the results of 13 genes obtained using DGE and qRT-PCR methodology.

The results of qRT-PCR were shown in Figure 6, the expression patterns of 13 genes showed a general agreement with the results of the DGE experiments, suggesting that DGE was an efficient and accurate strategy for the detection of differentially expressed genes.

## Discussion

The major goal of this study was to explore genome-wide gene expression profiles during the onset of lactation and to provide information for understanding the underlying molecular mechanisms. Three libraries were constructed from mammary tissues at three different stages (−35 d, −7 d and +3 d relative to parturition). Saturation analysis of the sequences indicated that the three libraries had reached saturation and were therefore complete assessments of all transcripts present in the libraries. The three libraries contained 192,928, 170,427 and 168,233 distinct clean tags, respectively. However, a reference database includes 35,945 transcripts. Theoretically, a tag should be generated by NlaIII digestion from the 3′-most end of a transcript. However, many transcripts contain more than one CATG site, so tags from other NlaIII sites were also generated in our libraries. Because only one tag can be generated per transcript from a given NlaIII site in a cDNA, these extra NlaIII tags represent genes redundantly present in the expression profile. Thus, the number of unique tags generated was greater than that of the annotated bovine genome.

To validate the DGE method, the levels of 13 genes were analyzed using qRT-PCR. Although the differences in the expression of some genes did not match the magnitude of those detected using the DGE method, the trends of upregulation and downregulation were similar. Compared with microarray and qRT-PCR methods, the sequencing method has been documented to be more sensitive for the estimation of gene expression, especially for low-abundance transcripts [17].

### Changes in mammary epithelial cells and the extracellular matrix at the onset of lactation

During the transition from pregnancy to lactation in the dairy cow, the mammary gland undergoes dramatic functional and metabolic changes, including the morphogenesis of mammary ducts during early pregnancy and differentiation of the mammary alveolus during late pregnancy [27], [28]. This study had found that many genes associated with the cell cycle were upregulated in stage I and downregulated in stage II, including cell division cycle associated 3 (CDCA3), Protein FAM83D (FAM83D), coiled-coil domain containing 99 (CCDC99), and others. These results suggested that cell proliferation occurred in late pregnancy and almost ceased after parturition, which was consistent with the early study [13]. Genes encoding extracellular matrix proteins were downregulated in stage I and upregulated in stage II. In addition, the ECM-Receptor interaction pathway was significantly enriched, and the differentially expressed genes in this pathway were downregulated and then later upregulated. These results demonstrated that, in stage I, the communication between cells and the extracellular matrix became weak with the proliferating of mammary epithelial cells and decreasing of the extracellular matrix, which recovered in stage II. A study in mice found that the communication between mammary epithelial cells and their environment became weak during the lactation period [11].

### Changes in genes associated with the immune response

At −7 d compared with −35 d, genes associated with the immune response accounted for 14.5% of differentially expressed genes, and genes associated with the defense response accounted for 8.20% of differentially expressed genes. Most of these genes were downregulated; for example, Chemokine (C-C motif) ligand 28 (CCL28) was completed turned off at −7 d. CCL28 is selectively expressed in certain mucosal tissues such as the exocrine glands, trachea, and colon and has a potent antimicrobial activity against *Candida albicans*, Gram-negative bacteria, and Gram-positive bacteria [29], [30]. Udder infections and mastitis are major problems for the dairy industry throughout the world, and the yearly costs are substantial. The risk of udder infection is the highest during the drying-off period and around the time of parturition [31], [32]. Adequate immune function is essential for the defense against udder infections. These genes associated with the immune response might be important for the immune function of the mammary gland and thus could be candidate genes for improving immune function.

### Expression profiles and regulation of milk protein genes

In this study, many milk protein genes were upregulated in stage I of lactogenesis. It has been shown previously in mouse [33], rat [34], and rabbit [35] that the expression of milk protein genes starts in early to mid-pregnancy, increases throughout pregnancy and reaches a plateau in late pregnancy and early lactation. However, Finucane et al [12] found that milk protein genes did not show significant changes in expression from late pregnancy to early lactation but that protein expression of glucose transporter GLUT1 did increase. The regulation of protein synthesis, particularly translation, in all mammalian tissues appears to be under control of the mTOR pathway [9]. Recently, studies in rodents and ruminants found that the mTOR pathway was very important for milk protein synthesis [36]–[40]. However, our results indicated that AKT (−1.10*) and PI3K (−1.20*) were downregulated significantly at −7 d compared with −35 d, which were upstream regulators of mTOR. In addition, some amino acid transporters were also downregulated during stage I, e.g. SLC43A2 (−1.85*), SLC3A2 (−1.58*) and SLC14A1 (−1.04*). In the mouse oocyte, mRNAs can be stored and then activated at the proper time [41]. Further study is required to investigate whether mRNA storage occurs in the bovine mammary gland.

The main regulator of milk protein expression in non-ruminant mammary glands appears to be the Jak-Stat5 signaling pathway [42]. In addition to protein synthesis, STAT5 is important for mammary gland development [43]. In bovines, STAT5 responds to prolactin and other lactogenic growth factors, and its activity increases during lactation [44], [45]. However, when compared with the rodent mammary gland, the role of bovine STAT5 in controlling milk protein expression through the Jak-Stat5 signaling pathway appears to be considerably weaker [46]. Our data appear to support a minor role of Jak-Stat5 signaling in milk protein synthesis, implied by the lack of any changes in the expression of PRLR and STAT5B during lactation. However, STAT5B activity is mostly regulated by its phosphorylation status, and this appears to be already regulated at the onset of lactation. Further study is required to investigate whether STAT5 phosphorylation increases at the onset of lactation.

### Lipid metabolism and its regulation at the onset of lactation

It has been speculated that there are two modes of regulation to control fatty acid synthesis in the mammary gland of the lactating mouse: the well-known SREBF1 system and a novel mechanism that acts at the posttranscriptional level in SCAP deletion mice fed a high-fat diet, leading to alterations to enzyme protein [47]. In this study, sterol regulatory element binding transcription factor 1 (SREBF1) and cofactor insulin induced gene 1 (INSIG1) increased 2.5-fold and 2.4-fold at −7 d compared with −35 d, respectively. It is possible that SREBF1 and cofactor INSIG1 regulate fat synthesis at the onset of lactation [48]. It has been postulated that, with the activation of secretion, SREBP-1 and its congener SREBP-2, the activators of cholesterol biosynthesis, are shuttled from the endoplasmic reticulum to the Golgi apparatus. Once in the Golgi, they are activated by proteolytic cleavage of a cytoplasmic fragment, a bHLH transcription factor that travels to the nucleus, directly activating the genes required for the synthesis of fatty acids and cholesterol [49]. This mechanism of action has been well studied in liver [50] and adipose tissues [51], and the promoters of the activated genes include SREs (sterol response elements) as well as binding sites for NF-Y, USF, SP1, and SP3 [52]–[54].

It is well known that the onset of lactation includes two different stages. And in stage I of lactogenesis, the mammary epithelial cells proliferate and differentiate, the extracellular matrix diminishes, and the communication between the two becomes weak. As milk protein genes and lipogenesis genes are upregulated, genes associated with the immune response are downregulated. In stage II of lactogenesis, the mammary gland restores the communication between mammary epithelial cells and the extracellular matrix. The main result of this process is the synthesis of milk using nutrients from the blood.

## Supporting Information

File S1 **The details of differentially expressed genes at −7 d compared with −35 d.**
(XLS)

File S2 **The details of differentially expressed genes at +3 d compared with −7 d.**
(XLS)

File S3 **The details of differentially expressed genes at +3 d compared with −35 d.**
(XLS)
